# Absence of Functional Leptin Receptor Isoforms in the POUND (Lepr^db/lb^) Mouse Is Associated with Muscle Atrophy and Altered Myoblast Proliferation and Differentiation

**DOI:** 10.1371/journal.pone.0072330

**Published:** 2013-08-14

**Authors:** Phonepasong Arounleut, Matthew Bowser, Sunil Upadhyay, Xing-Ming Shi, Sadanand Fulzele, Maribeth H. Johnson, Alexis M. Stranahan, William D. Hill, Carlos M. Isales, Mark W. Hamrick

**Affiliations:** Georgia Regents University (formerly Georgia Health Sciences University), Augusta, Georgia, United States of America; Georgia Regents University, United States of America

## Abstract

**Objective:**

Leptin receptors are abundant in human skeletal muscle, but the role of leptin in muscle growth, development and aging is not well understood. Here we utilized a novel mouse model lacking all functional leptin receptor isoforms (POUND mouse, Lepr^db/lb^) to determine the role of leptin in skeletal muscle.

**Methods and Findings:**

Skeletal muscle mass and fiber diameters were examined in POUND mice, and primary myoblast cultures were used to determine the effects of altered leptin signaling on myoblast proliferation and differentiation. ELISA assays, integrated pathway analysis of mRNA microarrays, and reverse phase protein analysis were performed to identify signaling pathways impacted by leptin receptor deficiency. Results show that skeletal muscle mass and fiber diameter are reduced 30–40% in POUND mice relative to wild-type controls. Primary myoblast cultures demonstrate decreased proliferation and decreased expression of both MyoD and myogenin in POUND mice compared to normal mice. Leptin treatment increased proliferation in primary myoblasts from muscles of both adult (12 months) and aged (24 months) wild-type mice, and leptin increased expression of MyoD and myogenin in aged primary myoblasts. ELISA assays and protein arrays revealed altered expression of molecules associated with the IGF-1/Akt and MAPK/MEK signaling pathways in muscle from the hindlimbs of mice lacking functional leptin receptors.

**Conclusion:**

These data support the hypothesis that the adipokine leptin is a key factor important for the regulation of skeletal muscle mass, and that leptin can act directly on its receptors in peripheral tissues to regulate cell proliferation and differentiation.

## Introduction

The cytokine-like hormone leptin is an important factor linking food intake with energy expenditure and body composition [1], [2]. Leptin is secreted from fat cells (adipocytes), but skeletal muscle is also a source of leptin [3], and serum leptin levels increase with increased muscle mass [4]. Leptin receptors have been identified in human skeletal muscle [5] and their expression is elevated with disuse atrophy [6] as well as with exercise [7]. On the other hand, leptin-deficiency increases expression of the muscle-wasting protein myostatin in myocytes [8], and the functional characteristics of skeletal muscle in leptin-deficient ob/ob mice have been noted to resemble those of aged rodents [9]. Perhaps not surprisingly, serum leptin and muscle mass were found to decrease with age in C57BL6 mice [10], but leptin treatment increased muscle mass and muscle fiber size in aged mice [11]. Furthermore, leptin-deficient ob/ob mice show decreased quadriceps muscle mass compared to normal, leptin-replete mice [12], [8], and caloric restriction in mice is associated with decreased serum leptin and decreased muscle mass [13]. Consistent with an anabolic role for leptin in the maintenance of skeletal muscle leptin treatment increases muscle mass in leptin-deficient ob/ob mice [14], [15] and decreases the expression of catabolic factors such as the ubiquitin ligases muscle RING-finger protein-1 (MuRF1) and atrogin-1 (MAFbx) [16].

Evidence from several mouse models implicates leptin in the regulation of muscle mass. The molecular mechanisms by which leptin exerts its effects in muscle do, however, remain unclear. Leptin treatment, either centrally or peripherally, increases circulating IGF-1 in leptin-deficient animals [15]. data also suggest that leptin may alter IGF-1 and musculoskeletal growth through growth hormone (GH)-independent pathways. For example, leptin treatment in fasting rodents increased GH but not IGF-1, whereas recombinant leptin therapy in fasting men and women increased IGF-1 but not GH [17], [18]. Thus, it is not clear whether leptin's anabolic effects on muscle are mediated solely through its effects on the GH-IGF1 axis, or via leptin's direct effects on peripheral tissues [19]. In ovo leptin injection increases muscle fiber size and muscle IGF-1 expression in the gastrocnemius of chickens [20], but leptin has also been observed to impair myogenesis in C2C12 cells and porcine myoblasts [21], [22]. As noted above, previous studies have detected elevated levels of IGF-1 with leptin treatment, but leptin deficiency is associated with elevated levels of myostatin (GDF-8), and myostatin deficiency has been found to increase circulating levels of liver-derived IGF-1 [23]. Thus, the relationship between leptin and IGF-1 is complex and complicated by the fact the leptin may alter both IGF-1 and myostatin signaling, and these two growth factors can both alter the same signaling pathways in skeletal muscle. Specifically, IGF-1's pro-hypertrophy effects on skeletal muscle fibers involve activation of the phosphoinositide 3-kinase (PI3K)/Akt signaling pathway, but myostatin can downregulate this same pathway [24], [25]. Leptin has previously been shown to activate PI3K/Akt signaling directly in whole skeletal muscle and isolated C2C12 cells [26], [27], and so the effect of leptin on muscle mass may be direct as well as mediated indirectly via IGF-1 and myostatin.

As noted above, the majority of earlier studies exploring the role of leptin in skeletal muscle have used either leptin-deficient ob/ob mice or established cell lines such as C2C12 cells; however, an impaired capacity for muscle regeneration has also been observed in leptin-receptor deficient db/db mice [28]. Here we employ two different mouse models to test the hypothesis that leptin can alter the proliferation and differentiation of myoprogenitor cells. First, in order to better determine the role of altered leptin signaling in skeletal muscle we utilized a new mouse model, the POUND (Lepr^lb/db^) mouse, which lacks all leptin receptor isoforms [29]. In contrast to db/db mice, which lack the long form of the leptin receptor but express the short receptor isoforms, POUND mice lack all functional leptin receptors and demonstrate greater weight gain and body mass compared to db/db mice [30]. we characterize the muscle phenotype of POUND mice, examine the capacity for proliferation and differentiation of primary myoblasts isolated from these mice, and investigate the signaling pathways that are altered in the absence of leptin signaling in muscle from POUND mice. also examine the effects of leptin treatment on proliferation and the expression of differentiation markers in primary myoblasts isolated from young adult (12 mo) and aged (24 mo) C57BL6 mice. Our data demonstrate that absence of leptin signaling in skeletal muscle induces muscle atrophy and fiber hypoplasia. This observed muscle atrophy is in turn associated with decreased levels of IGF-1 and elevated myostatin in skeletal muscle, and downregulated MAPK/ERK signaling and downregulation of Akt. Leptin, along with IGF-1 and myostatin, is therefore part of a complex endocrine and paracrine network regulating skeletal muscle mass.

## Materials and Methods

### Ethics Statement

All animal procedures were approved by the Institutional Animal Care and Use Committee of Georgia Health Sciences University (now Georgia Regents University).

### Mouse strains & Tissue Collection

POUND^lepr db/lb^ mice were purchased from Charles River laboratories at 12 weeks of age and were euthanized at 16 weeks of age. Animals were sacrificed by CO_2_ overdose and thoracotomy following procedures approved by the Institutional Animal Care and Use Committee of Georgia Health Sciences University. C57BL6 mice were purchased from the aged rodent colony at the National Institute on Aging, National Institutes of Health (USA) at 12 and 24 months of age and delivered to Georgia Health Sciences University, Augusta, GA. Animals were allowed to acclimate for approximately two weeks and were maintained at the Laboratory Animal Service Facility of the Georgia Health Sciences University.

Mice were weighed following sacrifice and the tibialis anterior dissected free and weighed. The soleus (SOL) and extensor digitorum longus (EDL) muscles were cut transversely along the middle of the muscle belly, embedded in OCT, snap frozen in liquid nitrogen, and cryostat sections stained with hematoxylin and eosin or with a Cy3-anti-laminin antibody (rabbit anti-mouse; Sigma L9393). Section images were captured and measured using ImageJ software.

### Primary myoblast culture and leptin treatment

Isolation of primary myoblasts of young and aged mice, and POUND mice, follows the general procedure described by Rando and Blau [31], [32] which we have recently used for myoblasts of aged and young mice [33]. Briefly, the left tibialis anterior muscle was dissected, weighed, placed in sterile PBS, and minced with a sterile scalpel under aseptic conditions. Minced muscle was digested in 0.2% collagenase type II (Gibco) and 1× trypsin. Upon completion of enzymatic digest, slurry was poured over a 70 µm cell strainer (Fisher) to remove any remaining connective tissue. The cells were then added to collagen type I (BD Bioscience) coated T-25 flasks. Primary myoblasts were allowed to attach for 72 hours. Cells were then maintained in proliferation medium (PM): DMEM (Hyclone) supplemented with 10% fetal bovine serum, 10% horse serum, 1% penicillin/streptomycin, and 0.5% chick embryo extract (Sera Labs U.K.). Medium was changed every 48 hours until T-25 flask was confluent. Once confluent, cells were trypsinized and counted using NucleoCounter (New Brunswick Scientific). Cells were then plated in a 96 well plate at 5,000 cells/cm^2^ and allowed to attach in proliferation medium for 48 hours. Proliferation medium was removed, cells washed with PBS, and DMEM supplemented with 1% insulin-transferrin-sodium selenite (ITS) was added followed by either vehicle in the case of POUND mice, control (PBS), low dose leptin (100 ng/ml) or high dose leptin (1000 ng/ml) in the case of cells from mice aged 12 and 24 months (R&D Systems, Minneapolis). After 24 h of treatment, MTS reagent was added according to the manufacturer's protocol (Promega, Madison) and absorbance at 492 nm was read 2 h later.

For differentiation assays, cells were isolated and cultured for one week until confluent as described above. Cells were then trypsinized and plated in 12 well plates at 5,000 cells/ml and allowed to attach overnight in proliferation medium. PM was removed, cells washed with PBS, and DMEM-supplemented with 1% ITS was added followed by the addition of leptin (100 ng/ml, 1000 ng/ml) in the case of 12 and 24 month old C57BL6 mice or vehicle in the case of POUND mice. Cells were maintained in treatment for 48 hour then harvested in TRIZOL® reagent (Invitrogen) for RNA isolation and subsequent cDNA synthesis (Bio-Rad). RT-PCR procedures are described below.

### RNA isolation, microarray processing, and signaling pathway analysis

Right tibialis anterior muscles were homogenized in Trizol reagent (Invitrogen Life Techonolgies, Carlsbad, CA USA) for microarrays. Total RNA was extracted using a standard chloroform protocol. For miRNA isolation, miRNeasy Micro kit (Qiagen, CA) was utilized. The microarray analysis was performed at the Georgia Health Science University Microarray Core facility. Briefly, mRNA was converted into double stranded cDNA using a T7-oligo (dT) promoter primer sequence and purified for use as a template for in vitro transcription. Transcription reactions were performed with T7 polymerase and biotinylated nucleotide analog/ribonucleotide mix for cRNA amplification. The biotinylated cRNA was prepared in the hybridization mix with oligonucleotide B2 and four control bacterial and phage cDNA. Labeled cRNA was hybridized to the Mouse 430 2.0 GeneChip array (Affymetrix) containing 45,000 probesets corresponding to 21,814 unique genes according to the manufacturer's instructions. Experiments were performed with two independent samples from the POUND mutants and control mice. We used Ingenuity Pathway Analysis (IPA) to decipher the possible biological relevance of gene expression changes established. (Ingenuity Systems, http://www.ingenuity.com website; Redwood City, CA, USA). Gene sets established by analysis of mRNA expression (significant expression changes), were subjected to IPA and significant pathways (p<0.05) were compared to each other. The genes and pathways showing highest predicted confidence were subjected to miRNA expression profile, revealing a mRNA-miRNA paired results.

### Quantitative RT-PCR (qRT-PCR)

1 ug of RNA from myoblast cultures or from muscle homogenates (soleus or extensor digitorum longus) was then utilized for cDNA synthesis. Reverse transcription was carried out using Quantitect reverse transcription kit (Qiagen, CA). The final cDNA products were used as the templates for subsequent RT-PCR. qRT-PCR was utilised to confirm the magnitude of expression changes of selected genes. Gene-specific primers (Table 1) were employed in qRT-PCR using Primer Assays (Qiagen). Concentrations of cDNA were adjusted to obtain good amplification. PCR was performed using an iCycler iQ™ instrument (Bio-Rad, Hercules, CA). A 20 µL PCR reaction contained 2 µL of diluted cDNA, 2.0 µL of gene specific primer and 10.0 µL of SYBR Green qPCR SuperMix-UDG. Cycle conditions were 95°C for 15 min and 40 cycles of 95°C for 15 s, 55°C for 30 s, and 72°C for 30 s. GAPDH and 18S were used for normalization. For miRNA quantitation, miScript reverse transcription kit was utilized for cDNA synthesis. The cDNA products were used as templates for RT-PCR. A 20 µL PCR reaction contained 2 µL of diluted cDNA, 2.0 µL of miRNA specific primer and 10.0 µL of SYBR Green SuperMix. Cycle conditions were 95°C for 15 min and 40 cycles of 95°C for 15 s, 55°C for 30 s, and 70°C for 30 s. RNU6-2 and SNORD95 were used for normalization. The difference between threshold cycles (ΔCT = CT Target Gene - CT Control Gene) is used in analysis. Fold change is calculated as 2-ΔΔCt where ΔΔCT = ΔCT Experimental condition - ΔCT Control.

**Table 1:** List of oligonucleotide primer sequences for qRT-PCR.

Name	Sequence	Amplicon Size
Leptin	Fwd: ACACACGCAGTCGGTATCCGC Rev: TCAGAATGGGGTGAAGCCCAGGA	74
Leptin receptor	Fwd: TGAGGTATCACAGGCGCAGCCT Rev: ACGCAGTTTTTGGGCTCAGACGT	76
Myogenin	Fwd: GGAAGTCTGTGTCGGTGGAC Rev: CGCTGCGCAGGATCTCCAC	150
MyoD	Fwd: GCCTGAGCAAAGTGAATGAG Rev: GGTCCAGGTGCGTAGAAGG	184

### Protein isolation, ELISA assays, and Reverse Phase Protein Analysis (RPPA)

Protein was isolated from the right extensor digitorum longus muscle of each mouse for ELISA and RPPA analysis. Muscles were placed in 1 mL phosphate buffered saline (PBS) and subjected to homogenization using Fisherbrand Tissuemiser® rotary homogenizer until large pieces of muscle were no longer visable. Samples were subjected to two freeze-thaw cycles to disrupt the plasma membrane then centrifuged briefly. Samples were separated into 250 µl aliquots and stored @ −80°C until assayed. IGF-1 ELISA kits were purchased from R&D Systems. Assays were performed according to manufacturer's protocol and samples were assayed without dilution. Myostatin ELISA kits were purchased from Alpco diagnostic and performed according to manufacturer's protocol as we have described previously (33).

For RPPA tissue lysates were two-fold-serial diluted for 5 dilutions (from undiluted to 1∶16 dilution) and arrayed on nitrocellulose-coated slide in 11×11 format. Samples were probed with antibodies by catalyzed signal amplification (CSA) and visualized by DAB colorimetric reaction. We stained 197 slides for 160 antibodies which were analyzed on ArrayPro then by supercurve R x64 2.15.1. There were 22 sets of replicated antibodies among 160 antibodies. performed QC test for each antibody staining (slide). QC score above 0.8 indicates good antibody staining. We only included in the data for the 160 individual antibodies with QC Scores higher than 0.80 in the Heatmaps. In the case of antibodies with replicates, the one with the highest QC Score was used. There were 37 individual mouse antibodies (labeled with “-M”), which were removed before creating heatmaps for the mouse xenograft samples. There were only 134 antibodies used on the heatmaps for xenograft samples. Slides were scanned on a flatbed scanner to produce 16-bit tiff image and spots from tiff images were identified and the density was quantified by MicroVigene. Relative protein levels for each sample were determined by interpolation of each dilution curves from the “standard curve” (supercurve) of the slide (antibody). Supercurve is constructed by a script in R written by Bioinformatics. These values (given as Log2 values) are defined as Supercurve Log2 (Raw) value”. The heatmap included was generated in Cluster 3.0 (http://www.eisenlab.org/eisen/) as a hierarchical cluster using Pearson Correlation and a center metric. The resulting heatmap was visualized in Treeview {http://www.eisenlab.org/eisen/) and presented as a high resolution.bmp format. Significant differences between groups were cross-validated using Western blots. Primary antibodies used were: p-AKT (1∶500 dilution, #9271 Cell Signaling) and p-MAPK (1∶500 dilution, #4370 Cell. Primary antibodies were incubated for 2 hrs, followed by washing and the application of secondary HRP-conjugated antibody (Santa Cruz). Immune complexes were visualized using the Novex chemiluminescent reagent (Invitrogen).

### Statistical analysis

Two-sample t-tests were used for comparisons between normal and POUND mice for body mass, tibial anterior mass, EDL, and MTS cell proliferation. To reduce the effect of outlying observations a two-sample test on the ranks of the data were used to analyze strain differences for the IGF-1 and myostatin ELISA assays and MyoD and myogenin ΔCT values. A one-way ANOVA was used to assess the effect of leptin treatment on normal mice for MTS cell proliferation, and ΔCT for MyoD and myogenin. Contrasts were constructed for comparison of leptin treatment (low dose leptin (100 ng/ml) or high dose leptin (1000 ng/ml)) to control. significance was determined at alpha = 0.05 and SAS© 9.3 (SAS Institute, Inc., Cary, NC) was used for all analyses.

## Results

### POUND mice are obese and show significant muscle atrophy

**Figure 1:**
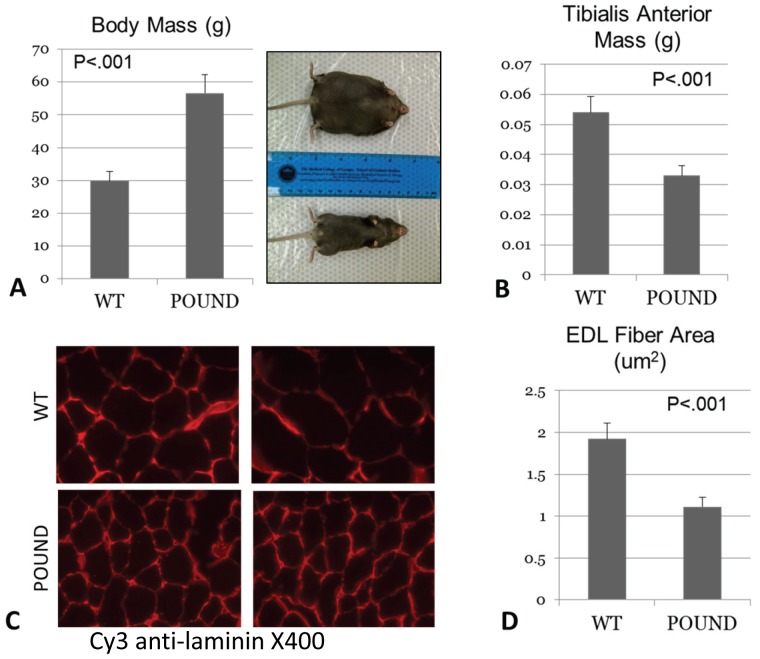
POUND mice show increased body weight but decreased muscle mass. A. Body mass of leptin receptor-deficient POUND mice is significantly greater than that of lean, wild-type (WT) mice (left panel). The image on the right shows the obese phenotype of a POUND mouse (top) compared with wild-type (bottom). B. Tibialis anterior muscle mass is significantly decreased in leptin receptor-deficient POUND mice. C. Frozen sections of the extensor digitorum longus muscle illustrate the larger muscle fibers and fiber cross-sectional areas of wild-type mice (WT, top row) compared to POUND mice (bottom row). D. Quantitative assessment of muscle fiber cross-sectional area demonstrates significantly decreased muscle fiber size in POUND mice. Mice are 16 weeks of age.

POUND mice are obese and weigh almost twice as much as control, lean mice (Fig. 1a). Tibalis anterior muscles of POUND mice are, however, reduced in mass approximately 35% compared to the larger muscles of lean, control mice (Fig. 1b). The significant reduction in muscle mass observed in POUND mice is due to a marked reduction in muscle fiber size, which is particularly evident in the fiber diameter of the predominantly fast-twitch extensor digitorum longus muscle (Fig. 1c, 1d). Muscle cross-sectional area at the middle of the EDL and SOL muscle belly is reduced in POUND mice, but muscle fiber number does not differ between groups, indicating that the atrophy in POUND mice is due primarily to reduced fiber size and not number (Table 2).

**Table 2:** Relative muscle cross-sectional area and fiber number (mean, S.D.) for the hindlimb muscles of POUND and WT mice.

Genotype	Muscle CSA (mm^2^ x e^−2^)	Fiber number (per mm^2^)
Extensor Digitorum Longus (EDL)		
POUND	1.85 (0.19)	16.2 (4.6)
WT	2.53 (0.42)	14.7 (6.2)
	*P<.001*	*P = 0.56*
Soleus (SOL)		
POUND	2.1 (0.59)	14.5 (2.9)
WT	2.45 (0.37)	13.7 (4.4)
	*P = 0.16*	*P = 0.66*

Fiber number is calculated by dividing muscle CSA by the average fiber cross-sectional area.

### Primary myoblasts from POUND mice show impaired proliferation and differentiation

**Figure 2:**
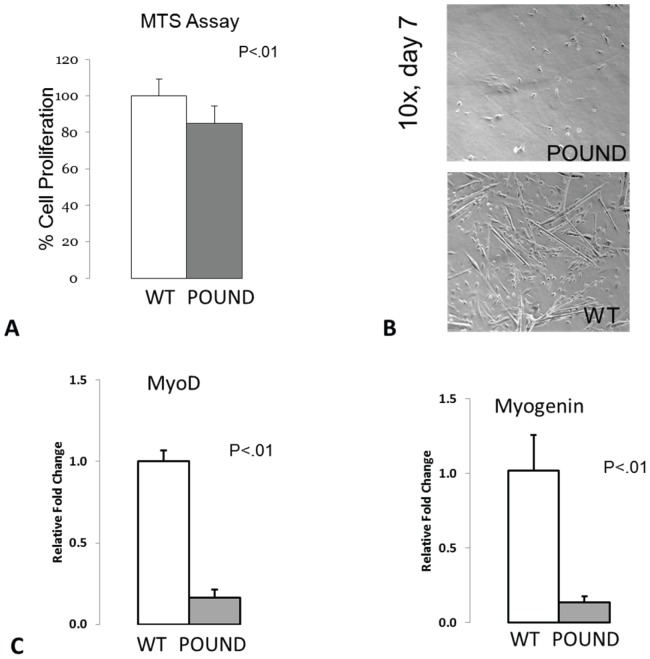
Primary myoblasts from POUND mice show impaired proliferation and differentiation. A. Primary myoblasts cultures from wild-type and POUND mice show a significant decrease in the proliferation and metabolic activity of myoblasts in POUND mice compared to normal wild-type mice as measured using MTS assay (left panel). B. Myoblasts from POUND mice (right panel, top micrograph) fail to differentiate normally and after 7 days do not develop into the elongate myotubes characteristic of normal, wild-type mice (right panel, bottom micrograph). C. Real-time PCR data show that that the early marker of myoblast differentiation, MyoD (left graph), and the later differentiation marker myogenin (right graph) are both significantly downregulated in myoblasts from POUND mice.

MTS assays performed on primary myoblasts isolated from the tibialis anterior muscles of normal mice and POUND mice show a significant decrease in metabolic activity, viability and proliferation in POUND mice (Fig. 2a). The impaired capacity for differentiation in POUND mice is further evident after 7 days in culture, where myoblasts from normal mice begin to form elongated myotubes but myoblasts from POUND mice retain a poorly differentiated morphology (Fig. 2b). The transcription factors MyoD and myogenin are expressed at the earlier and later phases of myoblasts differentiation, respectively, and both are significantly downregulated in cells of POUND mice (Fig. 2c, d).

### Leptin treatment stimulates myoblast proliferation in normal mice, but increases MyoD and myogenin expression only in myoblasts from aged mice

**Figure 3:**
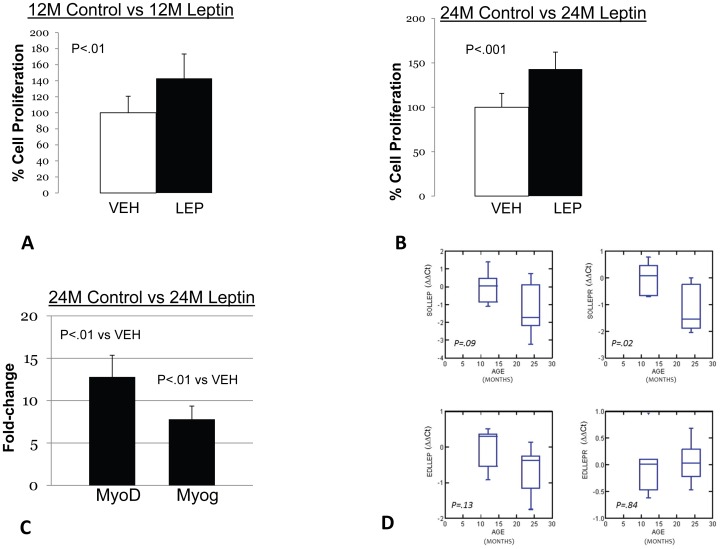
Leptin increases myoblast proliferation. A. Leptin-treatment (100 ng/ml) significantly increases cell proliferation and metabolic activity measured using MTS assay in primary myoblasts from mice 12 months of age. B. Leptin-treatment (100 ng/ml) also significantly increases cell proliferation and metabolic activity measured using MTS assay in primary myoblasts from mice 24 months of age. C. -treatment (100 ng/ml) significantly increases the expression of the myogenic factors MyoD and myogenin in primary myoblasts from mice 24 months of age. Leptin did not increase the expression of these factors in myoblasts from mice 12 months of age. D. Box-and-whisker plots showing ΔΔCt values (y-axis) for leptin (LEP) and leptin receptor (LEPR) expression in the soleus (SOL; top row) and extensor digitorum longus (EDL; bottom row) muscles of mice 12 and 24 months of age (x-axis). The whiskers mark the minimum and maximum values, the boxes the first and third quartiles, and the bar within the box indicates the median. Expression of the leptin receptor is not increased with age, and is significantly (P<.05) downregulated in aged soleus (SOLLEPR).

The impaired capacity for proliferation and differentiation that we observed in myoblasts from POUND mice led us to expect that leptin may have direct effects on primary myoblasts mediated via leptin receptors. We therefore treated primary myoblasts from aged (24 mo) and younger (12 mo) C57BL6 mice with leptin to determine leptin's effects on cell proliferation and differentiation. Leptin stimulated proliferation, as measured by MTS activity, in both young and aged myoblasts (Fig. 3a, b). This effect was significant at the lower (100 ng/ml) dose but the higher dose (1000 ng/ml) did not increase proliferation beyond what was observed at the lower dose (data not shown). The lower dose of leptin also increased expression of the differentiation markers MyoD and myogenin in aged myoblasts but not young myoblasts (Fig. 3c). PCR data (Figure 3d) do not show elevated levels of leptin receptor expression in aged versus young muscle, and in fact the receptor appears to be downregulated in aged soleus muscle tissue.

### Absence of leptin signaling in skeletal muscle impacts multiple signaling pathways including Akt and MAPK/ERK

**Figure 4:**
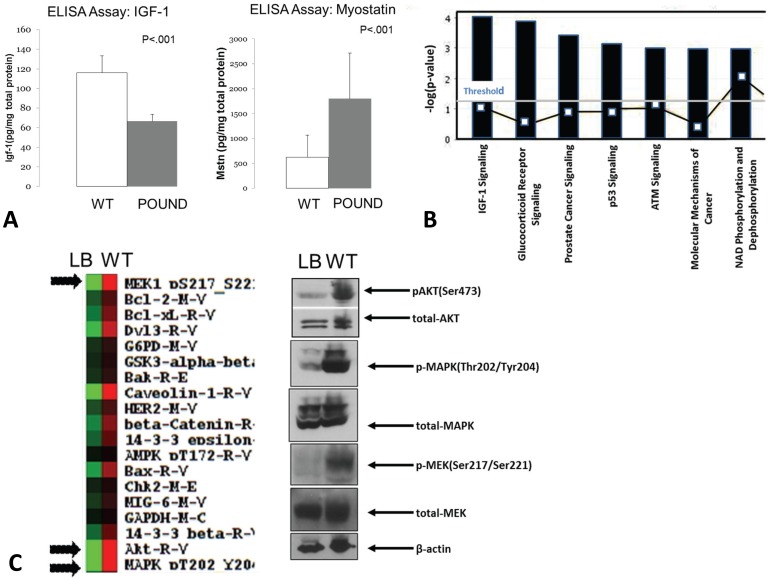
Altered leptin signaling in POUND mice alters IGF-1 signaling in skeletal muscle. A. ELISA assays show that muscle-derived IGF-1 is significantly decreased in the hindlimb muscles (extensor digitorum longus) from leptin receptor-deficient POUND mice (left graph), whereas protein levels of myostatin in hindlimb muscle are significantly elevated in POUND mice (right graph). B. Integrated pathway analysis from mRNA array comparing gene expression in tibialis anterior muscles of POUND mice with that of normal mice. The vertical axis represents the probability that a particular gene is associated with a specific canonical pathway by chance, the higher the score on this axis the lower the probability the association between gene and pathway is by chance alone. The strongest association revealed by the analysis is between genes altered in POUND mice and those associated with IGF-1 signaling. The open blue boxes connected by the lines represent ratio values indicating the ratio of genes detected in the pathway to the total number of genes in that particular pathway. C. Heat map from reverse phase protein analysis comparing protein expression in hindlimb muscle of POUND mice with that of normal mice. Arrows indicate proteins including Akt, MAPK, and MEK that are highly expressed in muscle from normal mice (red) but not highly expressed in muscle from POUND mice (green). Western blots shown on the right are for total and phosphorylated Akt, MAPK, and MEK.

ELISA assays demonstrate that muscle atrophy in POUND mice is associated with significantly lower levels of IGF-1 and elevated levels of myostatin (Fig. 4a). Pathway analysis of gene expression in whole-muscle tissue indicates that molecules associated with IGF-1 signaling are significantly altered in muscle from POUND mice compared to controls (Fig. 4b). Reverse phase protein array and western blots in turn show marked downregulation of Akt, MAPK, and MEK in muscle from POUND mice compared to normal, lean mice, and immunoblots of these same samples reveal decreased phosphorylated Akt-, p-MEK, and p–MAPK in POUND mice (Fig. 4c).

## Discussion

Regulation of food intake and energy expenditure by leptin is now well-documented, as are the signaling pathways mediated by leptin receptors in the hypothalamus [34]. It is also now becoming better appreciated that leptin receptors in peripheral tissues also play a key role in mediating leptin's effects in tissues such as bone and muscle [35], [36]. Indeed, it is now known that leptin receptors are highly expressed in skeletal muscle of vertebrates ranging from frogs [37] to humans [5], [38]. Thus, a functional role for leptin in skeletal muscle appears to be highly conserved across vertebrates. More specifically, a role for leptin as an anabolic, nutrient-induced growth factor that stimulates limb growth is indicated by experiments showing that exogenous leptin delivered peripherally increases limb outgrowth and promotes muscle development in both frogs and chickens [20], [37]. A role for leptin as a mitogen that induces cell proliferation is suggested by data showing that leptin treatment of leptin-deficient ob/ob mice stimulates expression of proliferating cell nuclear antigen (PCNA) and cyclin D1 in skeletal muscle [16], and increases proliferation of isolated mouse C2C12 cells [22]. Our data showing decreased cell proliferation in myoblasts lacking all forms of the leptin receptor are therefore consistent with these previous studies, as are our data indicating increased proliferation of primary myoblasts from 12 and 24 month mice in response to leptin treatment (100 ng/ml).

Hindlimb muscles of mice lacking functional leptin receptors show reduced mass and fiber size compared to those of lean mice, despite the fact that the mice lacking leptin receptors are almost twice the body weight of normal mice. The POUND mice are however relatively sedentary, and it is likely that lack of physical activity contributes to the reduced muscle mass observed in these animals. Exercise has, for example, been observed to increase lean mass in fatty Zucker rats [39]. Muscle atrophy in POUND mice is associated with a marked decrease in muscle fiber diameter, and not surprisingly primary myoblasts from POUND mice fail to differentiate normally and show reduced expression of the myogenic factors MyoD and myogenin compared to myoblasts from normal mice. These data suggest a role for leptin in myoblast differentiation and proliferation, despite the fact that certain studies suggest leptin may actually suppress the differentiation of C2C12 cells and porcine myoblasts [21], [22]. There is, however, some inconsistency in the reported effects of leptin on myoblast differentiation markers. For example, leptin was reported to decrease myogenin expression in pig myoblasts [21], however our data show myogenin was decreased in the absence of leptin receptors and myogenin expression was increased in myoblasts from aged mice. Moreover, while Pijet et al [22] reported that leptin impaired myogenesis, their data showed an increase in myogenin expression after 5 days of leptin treatment. One potential explanation for these contrasting findings is that we serum-starved our cells prior to leptin treatment since the serum itself contains leptin. It is possible that leptin treatment in the presence of serum actually represents a condition of hyperleptinemia, which may in turn have detrimental effects on myoblast differentiation.

While previous studies have documented reduced lean mass in mice lacking leptin [12], [15], [16], the data we present here provide new insights into the signaling pathways altered in skeletal muscle in the absence of leptin signaling, as well as the effects of age on the sensitivity of primary myoblasts to leptin treatment. Leptin is most well-known for activating Jak-Stat3 signaling in various cell types but leptin has also been shown to stimulate MEK/MAPK signaling in skeletal muscle [22]. Stimulation of MEK/MAPK by leptin has generally been thought to induce mitogenesis and proliferation by target cells, and our data are supportive of this model given the increase in myoblast proliferation with leptin treatment, decreased proliferation in POUND myoblasts, and lower MAPK and MEK protein levels in muscles from POUND mice. Our data also indicate that myogenic differentiation is impacted by leptin deficiency and leptin treatment. Specifically, myoblasts of mice lacking leptin receptors fail to differentiate normally and leptin treatment increases myoD and myogenin expression in myoblasts from aged mice. It is likely that the myogenic effects of leptin are mediated by crosstalk with myostatin and IGF-1, both of which are altered in POUND mice. IGF-1 is known to have multiple downstream effects in skeletal muscle including regulation of cell survival and myogenic differentiation through the PI3K/Akt pathway and stimulation of growth and proliferation through the MEK/MAPK pathway [40], [41]. Myostatin can also impact these effects of IGF-1 by repressing Akt activation, which in turn suppresses IGF-1 induced myogenesis [24], [42]. The increase in muscle-derived myostatin, decrease in IGF-1, and low levels of Akt in skeletal muscle of POUND mice are consistent with this model.

Our previous work showed that the C57BL6 mouse could be a useful model for studying age-associated pathologies of musculoskeletal system such as osteoporosis and sarcopenia [10]. Specifically, age-associated loss of muscle mass in this model is accompanied by a decline in circulating levels of leptin [10], and treatment with leptin increases the relative mass and fiber size of skeletal muscle in aged C57BL6 mice [11]. The in vitro data presented here reveal that leptin can stimulate the proliferative potential of aged myoblasts, which is subsequently accompanied by an increase in the levels of differentiation markers MyoD and myogenin. Notably myoblasts from aged mice showed a rise in MyoD and myogenin expression with leptin treatment whereas myoblasts from younger animals did not, even though there was no increase in leptin receptor expression in the aged mice. These data suggest that post-transcriptional mechanisms, such as microRNAs, may be involved in mediating age-associated changes in leptin signaling in skeletal muscle. Together, the in vivo results presented previously [11] and the in vitro data presented here suggest that aged myoblasts are indeed receptive and responsive to leptin. Moreover, signaling through the leptin receptor may be important in maintaining muscle mass, which declines with age in C57BL6 mice. As noted earlier, the functional characteristics of skeletal muscle in leptin-deficient ob/ob mice resemble those of aged rodents [9]. Leptin is therefore likely to be an important factor linking food intake with skeletal muscle anabolism and catabolism, and is also a factor that may be impacted with age-associated changes in food intake. Malnutrition is known to be common among older adults, and is associated with decreased appetite, reduction in food intake, and protein calorie deficiency [43]. The prevalence of malnutrition ranges from 15% among community-dwelling adults to more than 50% in the nursing home setting, suggesting that age-related declines in musculoskeletal performance are likely to be nutrient-related [44], [45]. It is possible that, in nutrient-depleted settings, recombinant leptin therapy may be one therapeutic strategy for improving muscle mass and musculoskeletal function in the aging population.
