# Correction: Absence of Functional Leptin Receptor Isoforms in the POUND (Lepr^db/lb^) Mouse Is Associated with Muscle Atrophy and Altered Myoblast Proliferation and Differentiation

**DOI:** 10.1371/annotation/3a7d6e24-137c-4603-93ca-879bec7fab80

**Published:** 2013-08-26

**Authors:** Phonepasong Arounleut, Matthew Bowser, Sunil Upadhyay, Xing-Ming Shi, Sadanand Fulzele, Maribeth H. Johnson, Alexis M. Stranahan, William D. Hill, Carlos M. Isales, Mark W. Hamrick

The corresponding author of the article was listed as the Editor. The correct Editor information is available below:

Rohit Kulkarni, Joslin Diabetes Center, Harvard Medical School, United States of America 

